# 

**Published:** 1914-06-06

**Authors:** 


### CONTENTS.

PAGE

**The Tuberculosis Society** ... ... ... ... ... 253

**The Poor-Law Medical Officers' Association** 254

**Hospital and Institutional News** ... ... ... 255

The Ulster Hospital Corps' Appeal—Our Hospital Sunday Appeal Number—National Medical Union Dinner—Prince Alexander on Committee Work—The Lost Liner: Medical Officer's Story—Retirement of a Liverpool Surgeon—The Cleansing of a Verminous Building—A New Development of the Pay-ward—The Pay-ward Movement in Jersey—Women's Hospital Burnt by Suffragettes—The Tendency of Tuberculosis Appointments—The Coming of Age of Medical Missions—The Future of Livingstone College—The New Superintendent at Benenden Sanatorium — The Salaries of Poor-Law Officers—Motor-car Cots—The General Medical Council and Death Certification—Scotland's First Homœopathic Hospital—A Picture Theatre as a Hospital Nuisance—Infirmary Superintendent as Sanitary Inspector—Southwark Guardians Appeal to Headquarters—Riff Pirates and London Hospitals—Death of an Ardent Non-Paneller: James Smith—The Whitsuntide Drug Market.

***Modern Methods Series*** ... ... ... ... 261

The Treatment of Frontal Sinus Suppuration.

[*See over.*]

### CONTENTS—(*continued*).

PAGE

**The Orthopædic Department** ... ... ... 262

VIII. The Plaster Room (*continued*).

**A Simple Treatment for Post-Partum Hæmorrhage** ... ... ... ... ... 264

**New Developments at "the London"** ... ... 265
How the Growth of Work is being Met.

**Round the World** ... ... ... ... ... 266

Fellow-Workers and their Doings.

**National Insurance Act Developments** ... ... 267
Prevention of Sickness under the Act.

**The Institutional Library** ... ... ... ... 269
A Practical Public Health Manual—A Lady's Attempt at a Text-book—The Cult of Tuberculosis—The Common Sense of Psycho-Analysis—A Pocket Guide to Ionic Medication.

**Personalia** ... ... ... ... ... ... 272

PAGE

**The Editor's Letter-Box** ... ... ... ... 271
The Retrograde Policy of the Bath Guardians—A List of Non-Panel Men—Poor-Law Medical Officers and *Post-Mortems*—Some Historic Children's Hospital Cots—Medical Hydrology.

**The Nursing and Care of the Sick Prior to 1850** ... ... ... ... ... ... ... 273
By Dr. F. M. SANDWITH, F.R.C.P., Gresham Professor of Physic, etc.

**The National Medical Union (Official)** ... ... 277
Report of Provisional Federating Council.
National Medical Union Dinner.

**Medical Appointments** ... ... ... ... 280

**The Institutional Worker** ... ... (Suppl.) 1
Hospital Supplies : Purchase by Contract or in Open Market?
Questions for June.
Questions Invited.
Service of Food to Night Nurses.

